# Zebrin II / Aldolase C Expression in the Cerebellum of the Western Diamondback Rattlesnake (*Crotalus atrox*)

**DOI:** 10.1371/journal.pone.0117539

**Published:** 2015-02-18

**Authors:** Joel W. Aspden, Carol L. Armstrong, Cristian I. Gutierrez-Ibanez, Richard Hawkes, Andrew N. Iwaniuk, Tobias Kohl, David J. Graham, Douglas R. Wylie

**Affiliations:** 1 Neuroscience and Mental Health Institute, University of Alberta, Edmonton, Alberta, Canada, T6G 2E9; 2 Department of Biology, Mount Royal University, 4825 Mount Royal Gate SW, Calgary, Alberta, Canada, T3E 6K6; 3 Lehrstuhl für Zoologie, Technische Universität München, Liesel-Beckmann Straße 4, 85354, Freising-Weihenstephan, Germany; 4 Department of Cell Biology & Anatomy, Genes and Development Research Group, and Hotchkiss Brain Institute, Cumming School of Medicine, University of Calgary, Calgary, Alberta, Canada, T2N 4N1; 5 Department of Neuroscience, Canadian Centre for Behavioural Neuroscience, University of Lethbridge, Lethbridge, Alberta, Canada, T1K 3M4; University of Tennessee Health Science Center, UNITED STATES

## Abstract

Aldolase C, also known as Zebrin II (ZII), is a glycolytic enzyme that is expressed in cerebellar Purkinje cells of the vertebrate cerebellum. In both mammals and birds, ZII is expressed heterogeneously, such that there are sagittal stripes of Purkinje cells with high ZII expression (ZII+), alternating with stripes of Purkinje cells with little or no expression (ZII-). The patterns of ZII+ and ZII- stripes in the cerebellum of birds and mammals are strikingly similar, suggesting that it may have first evolved in the stem reptiles. In this study, we examined the expression of ZII in the cerebellum of the western diamondback rattlesnake (*Crotalus atrox*). In contrast to birds and mammals, the cerebellum of the rattlesnake is much smaller and simpler, consisting of a small, unfoliated dome of cells. A pattern of alternating ZII+ and ZII- sagittal stripes cells was not observed: rather all Purkinje cells were ZII+. This suggests that ZII stripes have either been lost in snakes or that they evolved convergently in birds and mammals.

## Introduction

The gross anatomy of the cerebellar cortex varies considerably among vertebrates, ranging from a thin dome of cells in amphibians and snakes to a highly folded structure in mammals, birds, and some fish [1]. In mammals and birds, where the cerebellum can be divided into ten lobules, (referred to as "folia" in birds). Staining for certain molecular markers including zebrin II (ZII; = aldolase C [2]) reveals parasagittal stripes of high immunoreactivity (ZII+) interdigitated with stripes of little-to-no immunoreactivity (ZII-) (for reviews see [3–5]; for birds see [6–8]. The expression of ZII in sagittal stripes is congruent with other aspects of parasagittal cerebellar organization including mossy fibre input [9–17], climbing fibre input [18–23], Purkinje cell projections [24–26], and the physiological response properties of Purkinje cells [27–36].

ZII expression has been studied in the cerebella of numerous mammalian species [37–44] and a consistent pattern has emerged. In lobules VI-VII (a.k.a. the central zone), and ventral IX and X (nodular zone) ZII stripes are not apparent and most Purkinje cells are ZII+. However, lobules I-V (anterior zone), and lobules VIII and dorsal IX (posterior zone) consist of alternating sagittal ZII+ and ZII- stripes [45], [46]. It should be noted that although ZII stripes are not - observed in all lobules (i.e., central and nodular zones), other molecular markers are expressed in sagittal stripes in these lobules. For example, heat shock protein 25 and the human natural killer 1 antigen are expressed in sagittal stripes in the nodular zone [47], [48].

In birds, a strikingly similar pattern of the expression of ZII is apparent: alternating ZII+ and ZII- stripes are apparent in folia II-V and VIII and IX, whereas most Purkinje cells in folia VI, VII and X are ZII+ [6–8]. The only apparent difference between mammals and birds in the pattern of ZII expression is with respect to lobule I, which is striped in mammals, but uniformly ZII+ in birds (lingular zone) [6–8]. Given that the pattern of ZII expression is so similar in such disparate taxa, this could represent a homologous character and therefore also present in reptilian lineages. However, to the best of our knowledge, the only ‘reptile’ to be studied in this regard is a turtle (*Pseudemys scripta elegans*) [45]. The cerebellum of turtles is simple, consisting of a single unfolded dome [49], and the Purkinje cells are uniformly ZII+ [45]. Turtles are, however, a highly derived lineage with an uncertain relationship to extant diapsid reptiles and may not be representative of the expression pattern in lizards, snakes or crocodylians [50], [51]. In fact, it is possible that turtles are basal amniotes and that mammals are more closely related to sauropods than to turtles (see Fig. 1 of [52], [53]). No data are available for these other clades and therefore there is insufficient information to determine whether the ZII+/- stripe pattern in mammals and birds reflects homology or homoplasy. As a first step in resolving this question, we examined ZII expression in the cerebellum of the western diamondback rattlesnake (*Crotalus atrox*).

**Fig 1:**
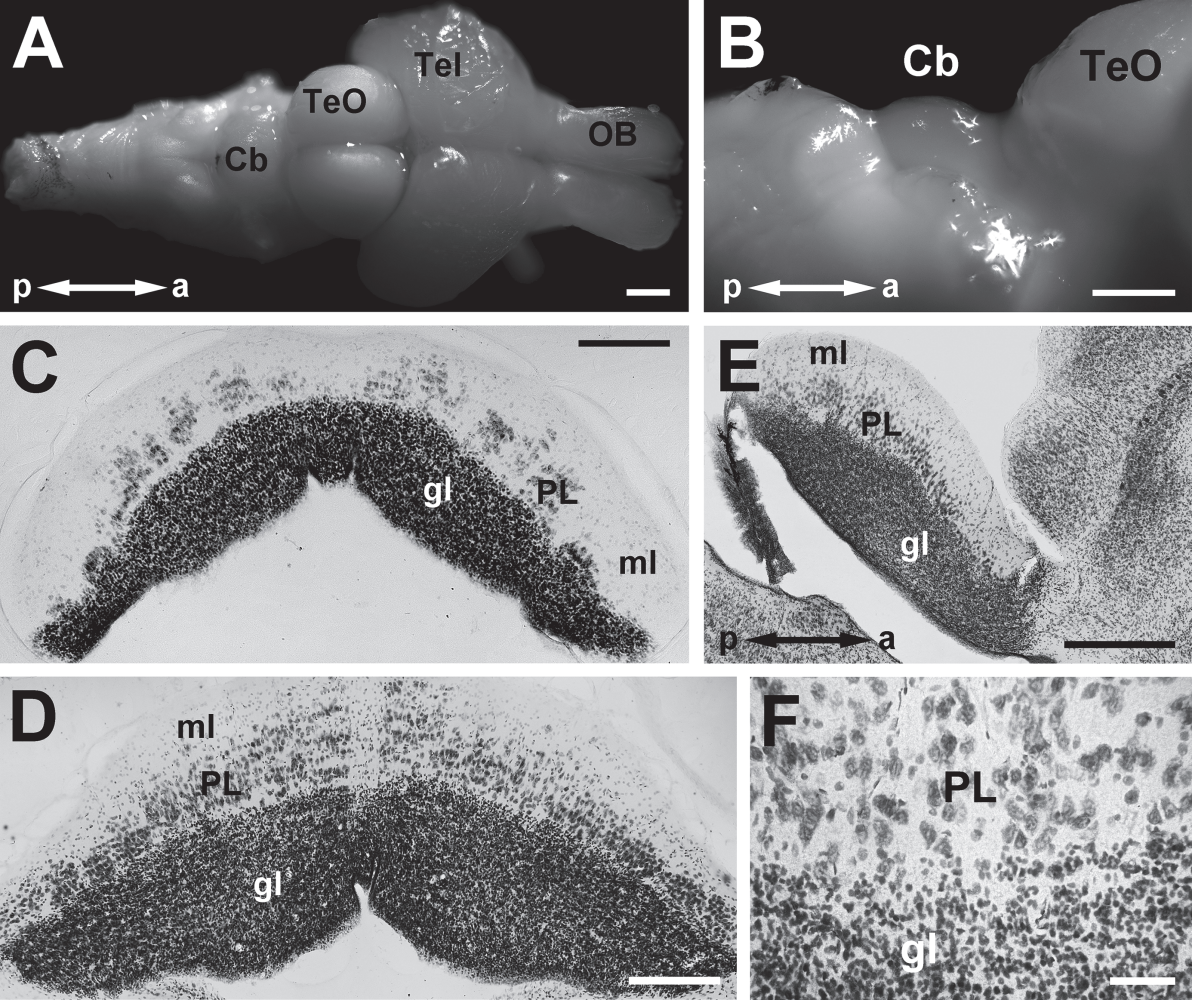
The cerebellum of *C*. *atrox*. **A** shows a dorsal view of the entire brain. **B** shows a lateral view at a higher magnification, highlighting the dome-shaped cerebellum (Cb) which is just caudal to the optic tectum (TeO). **C-E** are Nissl stained sections in the coronal (**C,D**) and sagittal planes (**E**) showing the Purkinje, granule and molecular layers (PL, gl, ml). **F** shows a higher magnification of the PL/gl border. Note that the Purkinje cells do not form a monolayer typical of mature birds and mammals. **C** shows a section in which the Purkinje cells form several clusters symmetrical about the midline. Other abbreviations; Tel = telencephalon; OB = olfactory bulb; a = anterior; p = posterior. Scale bars: 1mm in **A, B**; 250 μm in **C, D**; 500 μm in **E**; 50 μm in **F**.

## Materials and Methods

The brains of nine western diamondback rattlesnakes (*C*. *atrox*) were obtained from the Department of Zoology at the Technical University of Munich. Care, housing and sacrifice of the snakes followed the established guidelines for venomous snakes and was approved by the Regierung von Oberbayern (55.2–1–54–2532.6–9–12). Six of the snakes were young adults, approximately 2 years old (snout-to-ventral length ∼70cm, weight = 250–350 grams). The remaining three snakes were juveniles, approximately 10 months old (snout-ventral length = 20–40 cm, weight = 20–58 g). (With respect to the expression of ZII, we saw no differences between the cerebella of juveniles and adults). All snakes were bred in captivity (12 h: 12 h light: dark cycle, 22–30°C temperature range) in the Department of Zoology at the Technical University of Munich. The snakes were anesthetized with a combination of isoflurane and a mixture of ketamine hydrochloride (40 mg/kg i.m.) and xylazine hydrochloride (20 mg/kg i.m.) and perfused transcardially with 60 ml oxygenated ice-cold Ringer solution (in mM: 96.5 NaCl, 31.5 NaHCO_3_, 4 CaCl_2_, 2.6 KCl, 2 MgCl_2_ and 20 D-glucose, pH 7.4). The brains were removed and immersion fixed in 4% paraformaldehyde in phosphate buffer (PB; 0.1M, pH 7.4). Brains were then shipped to the Neuroscience and Mental Health Institute at the University of Alberta. The dura were removed and the brains were stored in 4% paraformaldehyde (in 0.1 M PB) for several weeks.

For two adult brains, whole mounts of the cerebella were immunostained using a protocol modified from that used for the mouse (*Mus musculus*) cerebellum [54]. The pia mater was removed and the entire snake brains were placed in Dent’s fixative [55] for 18hrs at 4°C, in Dent’s bleach [55] for 8 hours at 4°C and then dehydrated twice in 100% methanol at room temperature for 20 minutes each. The brains were then passed through 2–3 cycles of chilling to -20°C and thawing to room temperature in 100% methanol followed by overnight incubation in methanol at -20°C. The brains were rehydrated for 60 minutes each through 50% methanol, 15% methanol, and phosphate-buffered saline (PBS). After rinsing 3 X 10 minutes in PBS the brains were incubated in blocking buffer [56] for 6–8 hours at 4°C and then incubated for 48–96 hours in a mouse monoclonal ZII antibody produced by immunization with a crude cerebellar homogenate from the weakly electric fish *Apteronotus* [37]. The primary antibody was used directly from spent hybridoma culture medium diluted 1:10. The brains were then rinsed 3 X 30 minutes at 4°C, and incubated overnight at 4°C in peroxidase goat anti-mouse secondary antibody (1:100; Jackson Immunoresearch Laboratories, West Grove, PA). Finally, the brains were rinsed 3 X 3 hours each at 4°C in PBS followed by a final overnight rinse, incubated in 0.2% bovine serum albumin (BSA), 0.1% Triton X-100 in PBS for 2 hours at room temperature, and visualized with diaminobenzidine (DAB). A mouse cerebellum was processed in similar fashion as a positive control.

The other seven brains were equilibrated in a 30% sucrose solution (0.1 M PB), embedded in a gelatin, and serially sectioned at a thickness of 40μm. Sections were collected through the entire rostro-caudal extent of the cerebellum. Five of the brains were cut in the coronal plane, one was cut in the horizontal plane, and one was cut in the sagittal plane. All sections were collected in 0.1M PBS and divided into four alternate series. Sections from one series were Nissl-stained (cresyl violet) and the others were processed immunohistochemically to reveal ZII expression or combined ZII and calbindin (CB) expression. Sections were rinsed in wells containing 0.1 M PBS then incubated in blocking serum (10% normal donkey serum; Jackson Immunoresearch Laboratories) for one hour at room temperature. The sections were then incubated at 4°C for five days in 0.9% NaCl in 0.1 M PBS (pH 7.4) containing 0.1% Triton X-100 and an antibody to aldolase C (1:1000; goat-polyclonal; sc-12065, Santa Cruz Biotechnologies, Santa Cruz, CA). After five rinses in 0.1 M PBS, the sections were incubated for four hours at room temperature in Alexafluor-594 conjugated donkey anti-goat antibody (Jackson Immunoresearch Laboratories: diluted 1:100 in PBS, 2.5% normal donkey serum, and 0.4% Triton X-100). After the four hours, sections were rinsed five times in 0.1 M PBS, mounted on gelatinized slides, and briefly left to dry in the open air. For one series from each brain, we processed sections for both ZII and CB because all adult Purkinje cells express CB [57]. The above procedure was followed except, after the blocking step, the tissue was incubated in a solution containing both the anti-aldolase C and anti-calbindin (1:2,000; rabbit polyclonal, CB38, Swant) antibodies. The secondary to the anti-calbindin was a Alexafluor-488 conjugated donkey anti-rabbit secondary antibody (Jackson Immunoresearch Laboratories: diluted 1:200; in PBS, 2.5% normal donkey serum, and 0.4% Triton X-100). For negative controls, a section from each series was processed without the primary antibody. In addition, we processed a section from a pigeon (*Columba livia*) cerebellum with each series as a positive control.

### Microscopy and image analysis

Sections were viewed with a compound light microscope (Leica DMRE) equipped with the appropriate fluorescence filters for visualization. Images were acquired using a Retiga EXi FAST Cooled Mono 12-bit camera (QImaging) and analyzed with Openlab imaging software (Improvision). Photos were then stitched together in PTGui (New House Internet Services BV) for visualization of the entire sections. Adobe Photoshop (San Jose, CA) was used to adjust for brightness and contrast.

## Results

**Fig 2:**
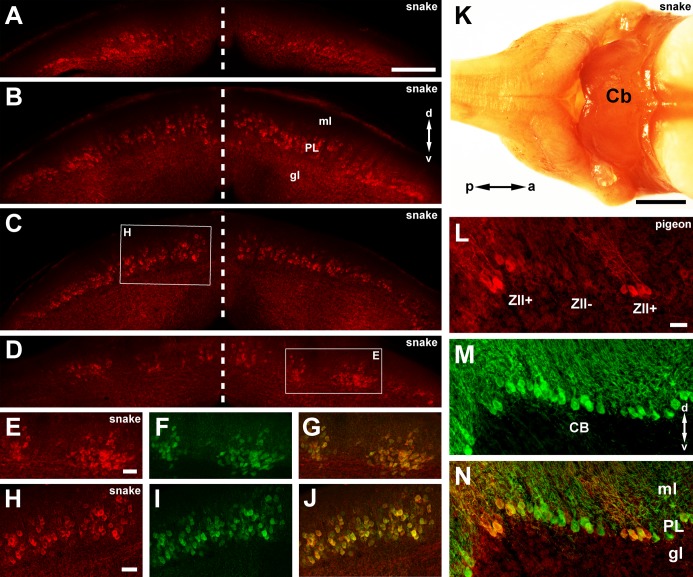
ZII expression in the cerebellum of the western diamondback rattlesnake. **A-D** shows photomicrographs of ZII (red) expression in serial coronal sections of the rattlesnake cerebellum (caudal to rostral). The dashed vertical lines indicate the midline. All Purkinje cells are ZII+. **E-G** shows a portion of the section in **D** (see inset) at higher magnification labeled for both ZII (red; **E**) and calbindin (CB; green; **F**). As seen in the overlay (**G**), all Purkinje cells are double-labeled. Similarly **H-J** shows a portion of the section in **C** at higher magnification labeled for both ZII and CB. **K** shows a dorsal view of the wholemount of the *C*. *atrox* brain that was stained for ZII. Note that the cerebellum is entirely ZII immunopositive. **L-N** shows a coronal section from a pigeon cerebellum processed for ZII (red; **L**) and CB (green; **M**). The overlay is shown in **N**. Other abbreviations; a = anterior, p = posterior, d = dorsal, v = ventral, gl = granule layer, PL = Purkinje Layer; ml = molecular layer. Scale bars: 250 μm in **A** (applies for **A-D**); 50 μm in **E** (applies for **E-G**), **H** (applies for **H-J**) and **L** (applies for **L-N**); 1mm in **K**.

The *C*. *atrox* cerebellum is unfoliated, and consists of a sheet of cells in a depression formed caudally by the swelling of the medulla and rostrally by the optic tectum (Fig 1A,B). In coronal sections and sagittal sections (Fig. 1C-E), the typical laminae of the cerebellum are clearly visible: the granular, Purkinje and molecular layers (gl, PL, ml). There is with a paucity of Purkinje cells at the midline (Fig. 1C,D). Note that the Purkinje layer is not an orderly monolayer as is typically observed in mature birds and mammals, but is several cells thick (Fig. 1F). This corroborates an early report by Steida [58] first who first noted that the Purkinje cells are found scattered throughout the molecular layer in snakes. In the anterior-most sections of the cerebellum (i.e. the anterior 100–150 microns of the cerebellum in adult snakes), the Purkinje cells appear as clusters separated by gaps containing no Purkinje cells (Fig. 1C; see also Fig. 2D-G). These clusters are symmetrical about the midline (Fig. 1C, 2D). It is unclear if the gaps between these Purkinje cell clusters are akin to the raphes observed in the developing cerebella of birds and mammals [59], [60].

ZII labelling was observed in Purkinje cell bodies, dendrites and axons. Fig. 2 shows immunostaining of the rattlesnake cerebellum for ZII expression. Unlike in birds and mammals, alternating ZII+ and ZII- stripes were not observed in the rattlesnake cerebellum, but rather all Purkinje cells expressed ZII to the same degree. Fig. 2K shows a picture of the rattlesnake cerebellum wholemount processed for ZII. Note the uniform expression in the cerebellum. This is clearly seen in a series of coronal sections (caudal to rostral) immunofluorescence-stained for ZII expression (Fig. 2A-D). Fig. 2E-J show higher magnification views of two transverse sections that are double immunofluorescence labeled for both ZII (red) and CB (green). Note that all Purkinje cells are double-labeled. In the most anterior sections (e.g., Fig. 2D), it may appear as if there are sagittal ZII+ and ZII- stripes, but this is because the Purkinje cells are in clusters separated by Purkinje cell-free gaps. This interpretation is confirmed in Fig. 2E-F, in which a section is double labeled for both CB and ZII. For comparative purposes, Fig. 2L-N shows a section through the cerebellum of a pigeon in which CB-immunopositive Purkinje cells can clearly be seen within a ZII- stripe.

## Discussion

Parasagittal stripes of alternating ZII immunoreactivity are not present in the cerebellum of the western diamondback rattlesnake, but rather all Purkinje cells are immunopositive. This is interesting given the alternating ZII+ and ZII- sagittal stripes in the avian and mammalian cerebella are strikingly similar. As discussed below, we suggest that the ZII stripe patterns in birds and mammals reflect homology, and that this was lost in turtles [45] and the western diamondback rattlesnake, and perhaps even other squamates.

### General Organization of the Reptilian Cerebellum

Whereas in birds and mammals the cerebellum is highly folded, in reptiles the cerebellum is a sheet or dome overlying the fourth ventricle (for review see [49]). The exception is crocodilians, where there are two transverse fissures that divide the cerebellum into three apparent lobes [49]. The basic cerebellar circuit, with two afferent systems (mossy and climbing fibres), a single output (Purkinje cells) and modulatory interneurons (stellate and Golgi cells), is much the same in birds, mammals and reptiles [61], [62]. Furthermore, like the cerebella of mammals and birds, the reptilian cerebellum has diverse sources of afferent input including climbing fibres from the inferior olive and mossy fibres from the spinocerebellar tracts, primary and secondary vestibular inputs, prepositus hypoglossi, trigeminal nuclei, raphe nuclei, etc., and the Purkinje cells project to the cerebellar and vestibular nuclei (see [62] for review).

It has been shown repeatedly in mammals and birds that the basic unit of cerebellar organization is the sagittal stripe [63]. Not only is this sagittal organization observed in the expression of molecular markers such as ZII [3–5], but also several other aspects of cerebellar anatomy and physiology including climbing and mossy fibre afferentation, Purkinje cell projections and Purkinje cell response properties [1], [33], [64–74]. Despite the fact ZII is not expressed in sagittal stripes in the cerebella of the rattlesnake or turtle, sagittal bands are observed with other aspects of cerebellar organization in reptiles. For example, in snakes, turtles and lizards, corticonuclear projections divide the cerebellum into four sagittal bands [75–77].

Thus, it appears that the organization of the cerebellum, from local circuitry to connectivity, is highly conserved among reptiles, birds and mammals [49], [62]. The exceptions are; (i) the degree of foliation and relative size of the cerebellum is much greater birds and mammals; and (ii) the pattern of alternating ZII+ and ZII- sagittal stripes is absent in the two reptiles that have been studied [45].

Although the presence of ZII stripes in the cerebella of birds and mammals, and their absence in turtles and snakes may indicate convergent evolution, the striking similarities in the pattern of the ZII+/- stripes in birds and mammals is suggestive of homology [6], [46]. As birds and mammals evolved from a common stem reptile, which also likely lead to other extant reptiles, this would imply that the alternating pattern of ZII+/- stripes was lost in snakes. This could have resulted from some rather simple changes. In both the nodular zone (ventral lobule IX and X in mammals, X in birds) and the central zone (lobules VI-VII), all Purkinje cells are ZII+ [45], [46]. Thus, perhaps the cerebellum of *C*. *atrox* cerebellum has been reduced to a nodular zone, or a central zone. Of these two possibilities, a reduction to nodular zone may be more plausible because the vestibulocerebellum (IXcd and X) is considered to be the most highly conserved [1], [49] and there are cerebellar projections to the vestibular nuclei in *Pseudemys* [75]. Further, the more rostral areas of the cerebellum that contain alternating ZII+/- sagittal stripes may have been lost in concert with the loss of the limbs. There is precedence for such an occurrence. In hummingbirds and caprimulgiform birds where there is a reduction in the size and utility of the hindlimbs, there is a dramatic reduction in the size of the anterior lobe [78], [79]. Larsell [49] noted that the cerebellum of the legless lizard *Anniella nigra* was “the smallest and simplest I have found among reptiles”. Black [80] also found that the size of the cerebellum became progressively smaller with degree of limblessness in Australian skinks. Thus, perhaps the ZII+/- stripes are absent in the snake cerebellum because it has been reduced to a nodular zone in concert with the evolution of limblessness. This would not explain the lack of ZII stripes in the turtle cerebellum although a reduction in the cerebellum could be associated with the reduction of axial musculature and evolution of the carapace [49].

As for a mechanism, the lack of ZII+/- stripes may occur as the result of a simple neoteny. In developing rats and mice, prior to the appearance of the ZII stripes, all Purkinje cells are ZII+ [81–84]. Thus, the global expression of ZII seen in the postnatal rat prior to the appearance of stripes, may be maintained in snakes if the progression beyond this stage to where the expression of ZII is suppressed in some Purkinje cells, does not occur.

## Conclusions

We showed that all Purkinje cells in the cerebellum of the western diamondback rattlesnake are ZII+, which contrasts with the pattern observed in mammals and birds where there are alternating sagittal ZII+ and ZII- stripes. As all Purkinje cells in the turtle are also ZII+ [45], and if the similarity of the pattern of ZII stripes in birds and mammals represents homology, this trait was lost in both snakes and turtles. Alternatively, the ZII stripes in birds and mammals may represent homoplasy, and the lack of ZII stripes in turtles and snakes represents the situation in stem reptiles. However, we must express caution in this regard as both turtles [85] and snakes [86] are highly derived and may not be representative of Reptilia. To further clarify the evolution of ZII stripes in the cerebellum it will therefore be necessary to examine the cerebella of crocodylians and lizards.
